# Microvascular Decompression for Trigeminal Neuralgia Caused by Venous Offending on the Ventral Side of the Root Entrance/Exit Zone: Classification and Management Strategy

**DOI:** 10.3389/fneur.2022.864061

**Published:** 2022-03-25

**Authors:** Wenhua Wang, Feng Yu, Sze Chai Kwok, Yuhai Wang, Jia Yin

**Affiliations:** ^1^Department of Neurosurgery, Shanghai Tenth People's Hospital, Tongji University, Shanghai, China; ^2^Department of Neurosurgery, 960 Hospital of PLA, Jinan, China; ^3^Shanghai Key Laboratory of Brain Functional Genomics, Key Laboratory of Brain Functional Genomics Ministry of Education, Shanghai Key Laboratory of Magnetic Resonance, Affiliated Mental Health Center (ECNU), School of Psychology and Cognitive Science, East China Normal University, Shanghai, China; ^4^Division of Natural and Applied Sciences, Duke Kunshan University, Kunshan, China; ^5^Shanghai Changning Mental Health Center, Shanghai, China; ^6^Department of Neurosurgery, 904 Hospital of PLA, Wuxi, China

**Keywords:** trigeminal neuralgia, root entrance/exit zone, microvascular decompression, offending artery, offending vein

## Abstract

**Background:**

Trigeminal neuralgia (TGN) is typically caused by an offending artery (OA) but may also involve an offending vein. Venous offending on the ventral side of the root entrance/exit zone (VO-VREZ) is particularly challenging.

**Objective:**

To analyze the rate and pattern of VO-VREZ and propose management strategy accordingly.

**Methods:**

VO-VREZ was classified into 3 types based on its anatomical relationship with a nerve root (A, the vein was covered by the nerve root entirely; B, the vein was lateral to the nerve root; and C, the vein penetrated the nerve root) and 3 groups based on the absence/presence of offending artery (I, no OA; II, suspected OA; and III, definitive OA).

**Results:**

The analysis included 143 cases with complete follow-up. Type A, B, and C accounted for 11.9, 31.5, and 56.6% of the cases, respectively. Group I, II, and III accounted for 24.5, 26.6, and 49.0%, respectively. Most group I VO-VREZ cases (26 out of 31) were managed with coagulation followed by division. Most group II VO-VREZ cases (31 out of 38) were decompressed with shredded Teflon interposition. Group III VO-VREZ was left in place in all 70 cases. Immediate pain relief was achieved in all cases. Temporary hemifacial hypesthesia occurred in 21 patients (14.7%), among which 14 were managed with Teflon decompression. Within the 4.5-year median follow-up, pain recurred in 11 patients (7.7%), but all with lesser intensity.

**Conclusion:**

VO-VREZ is not uncommon in patients with TGN. Different management strategy should be chosen according to the anatomical feature and the absence/presence of arterial conflict.

## Introduction

The incidence of trigeminal neuralgia (TGN) is ~3–5 per 100,000 individuals. In patients who do not respond to or could not tolerate pharmaceutical treatments, surgery is needed. TGN is typically caused by neurovascular conflict (NVC) (1). As such, microvascular decompression (MVD) is the most commonly used surgical modality for TGN (2) and recommended by most guidelines as the preferred surgical treatment for TGN and a variety of other cranial nerve diseases (3, 4).

The key to MVD is to identify the offending vessel and to achieve effective decompression of the nerve. The offending vessel is most commonly an artery (5), but has been reported to be a vein in 9.2–38.0% of cases (6). Offending veins tend to be more difficult to manage, with higher rate of treatment failure, recurrence, and complication after MVD (7–9).

Venous offending on the ventral side of root entrance/exit zone (VO-VREZ) is particularly challenging due to lack of direct view under the standard suboccipital retrosigmoid approach and multiple penetrating vessels that are prone to bleeding upon surgical maneuver. Another important reason for the technical difficulty in managing VO-VREZ is the limited efficacy of pre-operative assessment for offending veins in this region. Magnetic resonance imaging (MRI), particularly, T1-weighted imaging with gadolinium enhancement, is sensitive in revealing relatively large veins. Three-dimensional constructive inference in steady state (3D-CISS) could be used to reveal the veins in the cistern section or the veins that drain into the porus trigeminus of Meckel's cave due to the contrast by cerebrospinal fluid (CSF) (10, 11). For veins on the ventral side of the nerve root entrance/exit zone (REZ), however, the resolution of these imaging modalities is limited. We conducted a retrospective analysis to estimate the rate and the pattern of VO-VREZ, propose management strategy, and observe long-term follow-up outcomes.

## Methods

We retrospectively reviewed all cases of MVD for TGN conducted at our clinic during a period from January 1, 2011 to December 31, 2020. Diagnosis of TGN was established based on the criteria for classic TGN (13.1.1) of the International Classification of Headache Disorders 3 (ICHD-3). Some patients previously underwent other surgical treatments (e.g., percutaneous radiofrequency thermocoagulation, percutaneous balloon compression, or γ-knife surgery). As part of the routine clinical management of TGN, preoperative time-of-flight sequence MRI examination was performed in all patients. The analysis included all cases in which one or more veins inside of the nerve REZ were found to be in close contact with the nerve root and considered as potential offending vessels in the MVD surgery. The current study was approved by the Ethics Committee at author's hospitals. Data are anonymous, hence informed consent was not applicable.

Microvascular decompression surgery was conducted via a standard suboccipital retrosigmoid approach. After releasing CSF under the microscope, the cerebellar hemisphere was retracted. The arachnoid membrane between the petrosal vein and the facial-auditory nerve was opened and the cisternal segment of the trigeminal nerve was exposed. The entire length of the trigeminal nerve root (from the pons to the entrance of Meckel's cave) was dissected. Offending arteries were managed routinely by the Teflon tap. The pattern and management of VO-VREZ are described in the Results Section.

Follow-up was conducted at 3, 6, and 12 months, and every 1 year afterwards. These included routine physical examination and facial sensory testing in majority of the cases. Online interview was conducted if patients failed to visit our clinic at the scheduled time. Outcome measures included initial pain relief, pain recurrence, and any type of sensory disturbance as assessed by the Barrow Neurological Institute (BNI) pain intensity score and the facial numbness score (12). Complete pain relief was defined as BNI pain score I. Partial pain relief was defined as BNI pain score II or III. Treatment failure was defined as BNI pain score IV or V.

## Results

A total of 671 cases of MVD for TGN were screened. VO-VREZ was identified in 154 cases (64.32 ± 9.46 years of age, range: 27 to 84; 95 women). The mean disease duration was 5.5 years (range: 0.5–20). Demographic and clinical characteristics of the patients with VO-VREZ are shown in Table 1. The MRI examination identified VO-VREZ in 21 out of the 154 cases. Eleven patients were lost to follow-up, and the analysis included 143 patients. No major complications (i.e., infection, cerebrospinal fluid leakage, and/or intracranial hemorrhage) occurred during the peri-operative period. Immediate complete pain relief was achieved in all 154 cases after the surgery.

**Table 1:** Demographic and clinical characteristics of the patients with VO-VREZ.

		***n* = 154**
Female sex, *n* (%)		95 (61.7%)
**Age (y)**
	≤ 39	8 (5.2%)
	40–49	12 (7.8%)
	50–59	36 (23.4%)
	60–69	51 (33.1%)
	70–79	40 (26.0%)
	≥80	7 (4.5%)
Left side		71 (46.1%)
**Divisions involved**
	V1	7 (4.5%)
	V2	26 (16.9%)
	V3	29 (18.8%)
	V1 + V2	14 (9.1%)
	V2 + V3	53 (34.4%)
	V1 + V2 + V3	25 (16.2%)

**Figure 1:**
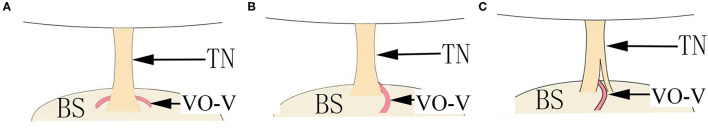
Three types of venous offending on the ventral side of the root entrance/exit zone (VO-VREZ) based on anatomical relationship of the vein and the trigeminal nerve root entrance/exit zone. **(A)** The full route of the vein was covered by the root entrance/exit zone; **(B)** The vein went out laterally from the root; **(C)** The VO-VREZ ran through the root. TN, trigeminal nerve; BS, brain stem; VO-V, venous offending on the ventral side of root entrance/exit zone.

Based on the anatomical relationship between the offending veins and the nerve root, 3 types of VO-VREZ were identified: type A when the entire route of the vein was covered by the nerve root (17/143, 11.8%); type B when the vein was lateral to the nerve root (45/143, 31.5%); and type C when the vein penetrated the nerve root (81/143, 56.6%) (Figures 1A–C). According to the presence/absence of offending artery in the NVC, VO-VREZ was classified into 3 groups: I (35/143, 24.5%), no OA; II (38/143, 26.6%), suspected OA (when a offending artery was in contact with the root but without any visible indentation or intermittent contact with nerve root with CSF pulsation); and III (70/143, 49.0%), definitive OA (displacement and/or a distortion of the root, and marked indentation of the nerve root) (13).

**Figure 2:**
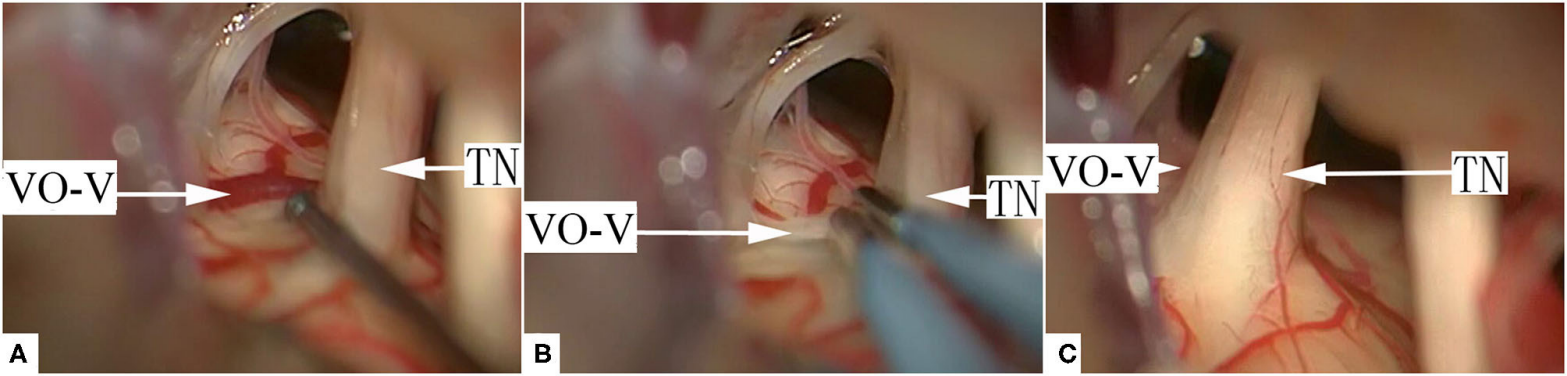
A representative case of typical group I, type A VO-VREZ. **(A)** The full route of the vein was covered by the nerve root entrance/exit zone. **(B)** The VO-VREZ was electrocoagulated. **(C)** The trace of VO-VREZ after electrocoagulation and division. TN, trigeminal nerve; VO-V, venous offending on the ventral side of root entrance/exit zone.

**Figure 3:**
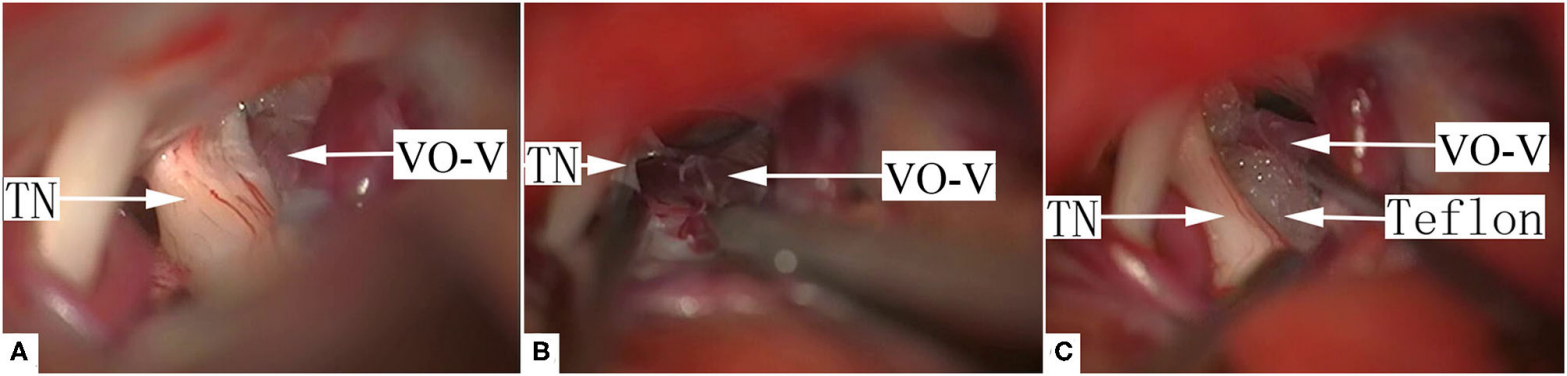
A representative case of group I, type B VO-VREZ with relatively large diameter. **(A)** The VO-VREZ was lateral to the nerve root. **(B)** The VO-VREZ was dissected. **(C)** The nerve root was decompressed with shredded Teflon interposition. TN, trigeminal nerve; VO-V, venous offending on the ventral side of root entrance/exit zone; Teflon, shredded Teflon interposition.

**Figure 4:**
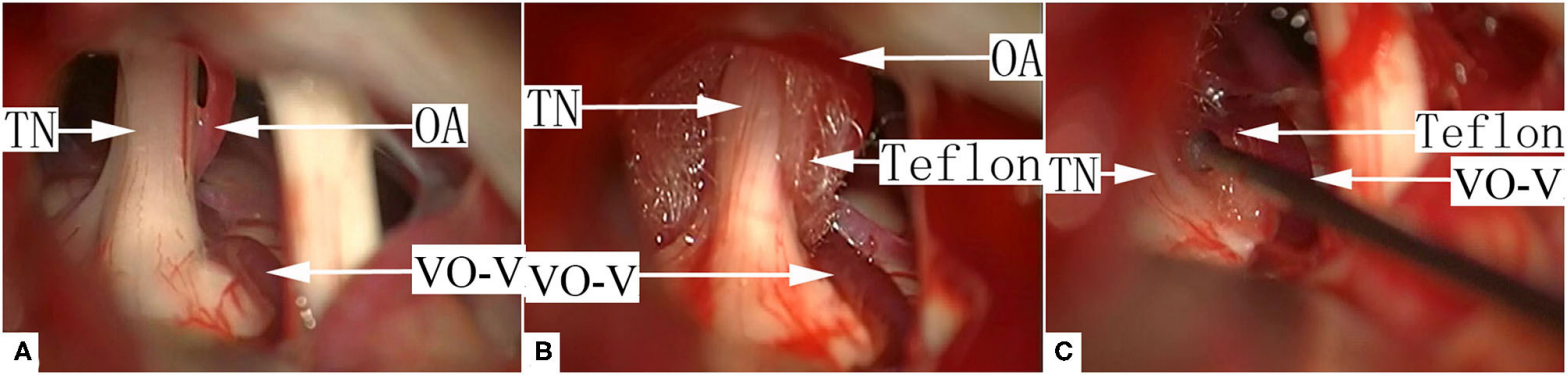
A representative case of typical group II, type B VO-VREZ. **(A)** The VO-VREZ was lateral to the nerve root and a suspected offending artery was found. **(B)** Decompression of the offending artery with shredded Teflon interposition. **(C)** Dissection and decompression of VO-VREZ with shredded Teflon interposition.TN, trigeminal nerve; VO-V, venous offending on the ventral side of root entrance/exit zone; OA, offending artery.

**Figure 5:**
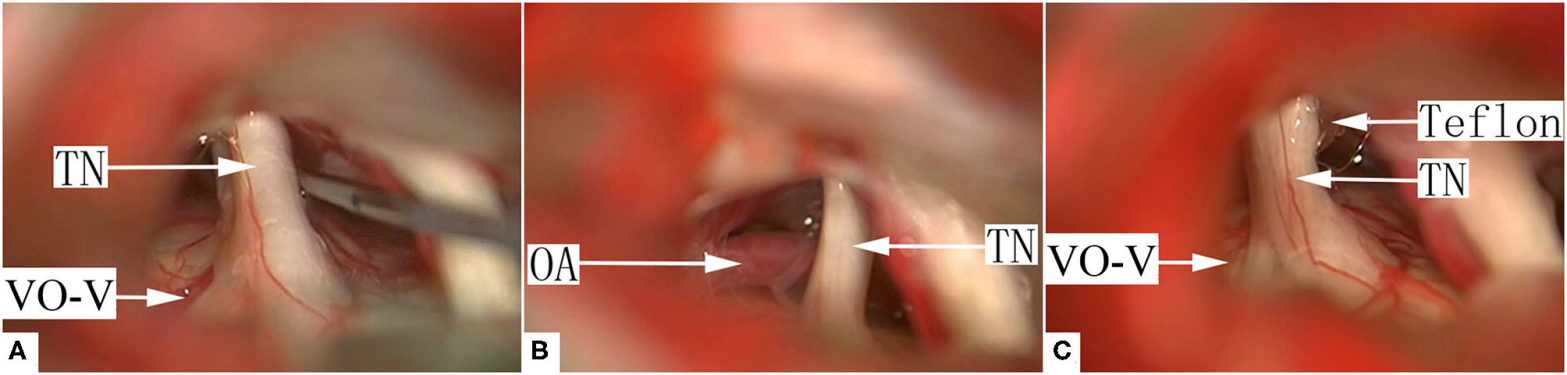
A representative case of group II, type A VO-VREZ. **(A)** The whole length of the vein was covered by the nerve root. **(B)** There was a suspected offending artery behind the trigeminal nerve. **(C)** The suspected offending artery was decompressed by a shredded Teflon interposition. VO-VREZ was electrocoagulated and then divided. TN, trigeminal nerve; VO-V, venous offending on the ventral side of root entrance/exit zone; Teflon, the shredded Teflon interposition.

**Figure 6:**
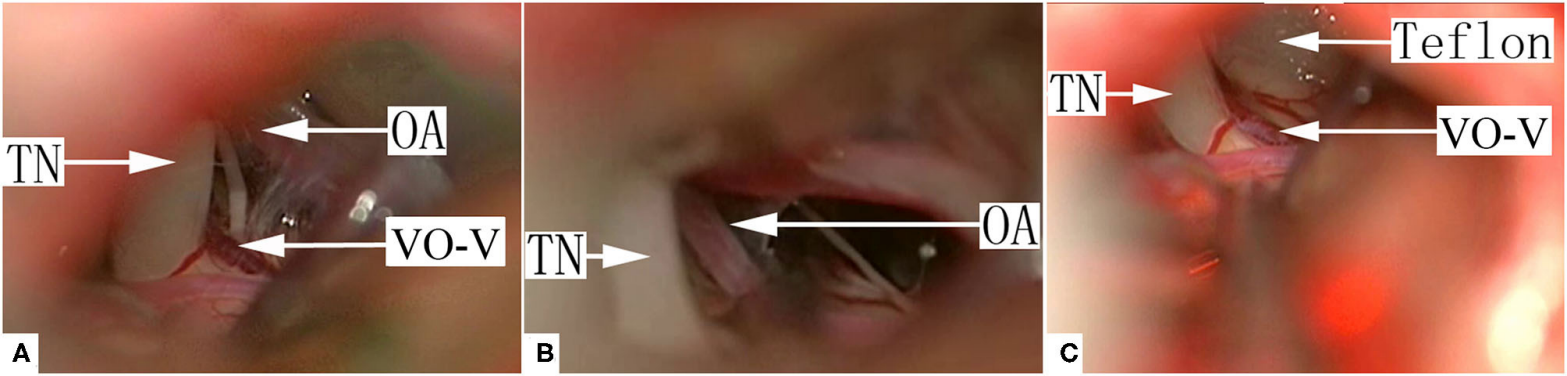
A representative case of group III, type C VO-VREZ. **(A)** A definitive offending artery was identified. The small VO-VREZ ran through the trigeminal nerve root. **(B)** The offending artery was dissected. **(C)** The nerve root was decompressed from the artery with shredded Teflon interposition. The VO-VREZ was left alone. TN, trigeminal nerve; OA, offending artery; VO-V, venous offending on the ventral side of root entrance/exit zone; Teflon, shredded Teflon interposition.

Since the VO-VREZ represents the only possible culprit vessels, most cases of group I VO-VREZ (26/35) were managed with coagulation followed by division (Figures 2A–C, Supplementary Video 1). In the remaining 9 cases, the offending veins had relatively large diameters (>2 mm) and were managed with decompression with shredded Teflon interposition to avoid jeopardizing blood circulation to the brain stem and cerebellum (Figures 3A–C, Supplementary Video 2). Among the 38 cases of group II VO-VREZ, 31 were managed by decompression with shredded Teflon interposition (Figures 4A–C, Supplementary Video 3). The remaining 7 cases were managed with electrocoagulation, followed by division in 4 cases due to bleeding upon dissection attempt and division because of the limited vision in 3 cases (type A) (Figures 5A–C, Supplementary Video 4). In the 70 cases of group III VO-VREZ, the veins seemed unlikely represents culprit vessels and were left alone for 2 reasons (Figures 6A–C, Supplementary Video 5): (1) majority of the veins are small in diameter; and (2) there were definitive OA. In group II and III VO-VREZ, the OA was treated with decompression with shredded Teflon interposition.

**Figure 7:**
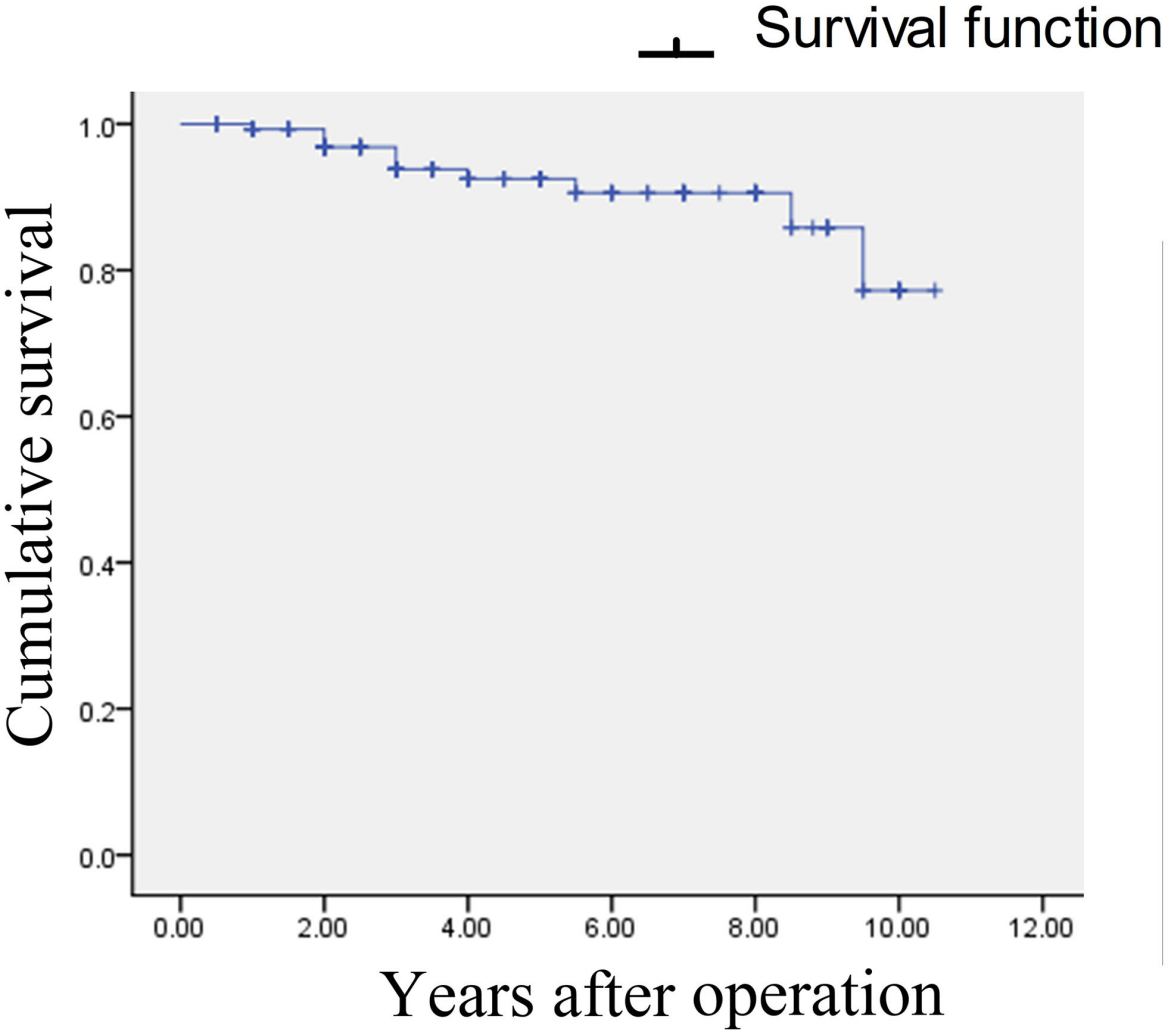
Kaplan–Meier survival analysis. Pain recurred in 11 patients.

No major complications (i.e., infection, cerebrospinal fluid leakage, and/or intracranial hemorrhage) occurred during the peri-operative period. Hemifacial hypesthesia was reported by 21 patients (14.7%) after MVD (details in Table 2), but most of them dissipated within 3–6 months. Within the 4.5 years of follow-up (range: 0.5–10.5), 11 patients (7.7%) reported pain recurrence with the BNI pain score at either II or III. Pain free survival was defined as the time to the recurrence of facial pain after surgery. The Kaplan–Meier curves were constructed based on pain free survival (Figure 7). The pain was well controlled by small-dose analgesics in 7 cases. Four patients were treated by percutaneous balloon compression 2–5 years postoperatively. Classification, management strategy, complication, and recurrence are shown in Table 3.

**Table 2:** The rate of postoperative hemifacial hypesthesia in patients with VO-VREZ of distinct types and groups.

		**Hemifacial hypesthesia**	***P*-value**
**Type**
	A	5.9% (1/17)	
	B	26.7% (12/45)	0.024
	C	9.9% (8/81)	
**Group**
	I	5.7% (2/35)	
	II	42.1% (16/38)	<0.001
	III	4.3% (3/70)	
**Management**
	Division	12.1% (4/33)	
	Decompression	35.0% (14/40).	<0.001
	Not intervened	4.3% (3/70)	

*Statistical comparison was conducted using Fisher exact test*.

**Table 3:** Management strategy for VO-VREZ of distinct types and groups.

	**Division *n* = 33**	**Decompression *n* = 40**	**Not intervened *n* = 70**
**Group I**, ***n*** **=** **35**
Type A, *n* = 8	8 (0/1)		
Type B, *n* = 12	7 (0/1)	5 (1/1)	
Type C, *n* = 15	11 (1/1)	4	
**Group II**, ***n*** **=** **38**
Type A, *n* = 6	3	3 (1/0)	
Type B, *n* = 23	4(3/0)	19 (8/2)	
Type C, n=9		9 (4/0)	
**Group III**, ***n*** **=** **70**
Type A, *n* = 3			3
Type B, *n* = 10			10 (0/1)
Type C, *n* = 57			57 (3/4)

*The numbers in parentheses are number of patients with postoperative hemifacial hypesthesia/recurrence*.

## Discussion

The current study showed that VO-VREZ is not rare in patients with TGN (23.0%; 154/671). VO-VREZ could be classified into 3 groups: I, with no OA; II, with suspected OA; and III, with definitive OA. Group I-III accounted for 24.5%, 26.6%, and 49.0% of the cases, respectively. Group I VO-VREZ was primarily managed with electrocoagulation followed by division, while in the few cases with >2 mm vein diameter, the offending veins were managed with decompression with shredded Teflon interposition. Group II VO-VREZ was managed by decompression with shredded Teflon interposition unless upon bleeding during dissection or limited vision and expected difficulty in dissection (type A). Group III VO-VREZ was mostly of small veins of type C and were left alone. No major complications (i.e., infection, cerebrospinal fluid leakage, and/or intracranial hemorrhage) occurred during the peri-operative period. Immediate complete pain relief was achieved in all 143 cases after the surgery. Hemifacial hypesthesia occurred in 21 patients (14.7%), and, seemingly, more frequently in patients with group II and type B VO-VREZ receiving decompression with shredded Teflon interposition. The recurrence rate was only 7.7%. We did not attempt to analyze the relationship between recurrence and VO-VREZ type due to the relatively small sample size.

Because offending veins in TGN are more difficult to manage (14, 15), it is one of the major reasons for treatment failure, recurrence, and complications in MVD surgery (16, 17). The report of these cases have been increasing, mostly as case series (18, 19), but there were few descriptions about VO-VREZ. The recurrence rate within the 6.5 years follow-up was 7.7% in the current study. Such a rate is seemingly lower than previously reported (20). The main reason we believed was to pay more attention to the venous offending, such as VO-VREZ. According to their different pattern, a different management strategy was chosen. The VO-VREZ, reported firstly, was included in the current study, whereas past studies included offending veins along the entire length of the trigeminal nerve root.

For offending veins with relatively large diameter, most neurosurgeons choose to avoid destroying the veins. For veins of smaller size, coagulation followed by division is more often used. For VO-VREZ, the close proximity to the brainstem and lack of direct vision represents added complexity. Based on a study by Rhoton (21) showing extensive collateral circulation in posterior cranial fossa, we propose that VO-VREZ should be managed with different approaches under different circumstances. Specifically, a more aggressive approach should be taken to maximize therapeutic efficacy if TGN could be attributed to the offending vein with certainty (group I VO-VREZ). If a definitive offending artery is identified (group III VO-VREZ), more conservative approach should be taken. For group II VO-VREZ, we chose the standard decompression with shredded Teflon interposition if possible. Other factors that were considered in the management included size of the offending veins, the number of penetrating vessels, and intraoperative bleeding. The outcomes in the current study, including the safety profile and recurrence, supported varying management strategies based on the presence of absence of either suspected or definitive offending artery.

Surgical Points:

1. During the electrocoagulation procedure, the tip width of the bipolar electrocoagulation tweezers was 0.1 mm, and the output of the electrocoagulation power was minimized. The trunk of the vein, nerve, or brain stem surface should not be damaged.
2. Division of the veins should be performed gently when the VO-VREZ adheres to the brain stem. VO-VREZ branches that adhere to the surface of the brain stem should be individually cut after electrocoagulation.

A major limitation of the current study is its retrospective nature. Management of individual cases was not guided by a fully formulated strategy prior to surgery. Also, the follow-up period is relatively short. As a result, the recurrence might have been underestimated.

## Conclusion

Venous offending is not rare in TGN patients. We propose to classify VO-VREZ into A/B/C types based on the anatomical relationship between VO-VREZ and the trigeminal nerve root, and into I/II/III groups based on the presence/absence of offending arteries. Group I VO-VREZ (with no OA) should be electrocoagulated and then divided. Group II VO-VREZ (with suspected OA) should be decompressed using shredded Teflon interposition. Group III VO-VREZ are not culprit vessels unless indentation is notified on the nerve root and should be left in place.

## Data Availability Statement

The raw data supporting the conclusions of this article will be made available by the authors, without undue reservation.

## Ethics Statement

The studies involving human participants were reviewed and approved by Shanghai Tenth People's Hospital. Written informed consent for participation was not required for this study in accordance with the national legislation and the institutional requirements.

## Author Contributions

JY and FY performed the surgery. WW and YW analyzed the data. SK contributed to manuscript writing. All authors read and approved the final manuscript.

## Funding

This work was supported, in part, by the National Natural Science Foundation of China (#81671201 to JY and #81871598 to YW).

## Conflict of Interest

The authors declare that the research was conducted in the absence of any commercial or financial relationships that could be construed as a potential conflict of interest.

## Publisher's Note

All claims expressed in this article are solely those of the authors and do not necessarily represent those of their affiliated organizations, or those of the publisher, the editors and the reviewers. Any product that may be evaluated in this article, or claim that may be made by its manufacturer, is not guaranteed or endorsed by the publisher.

## Supplementary Material

Supplementary Video 1 A representative case of typical group I, type A venous offending on the ventral side of the root entrance/exit zone (VO-VREZ). Exploration of the entire trigeminal nerve root (from the brainstem exit to the porus trigeminus of Meckel's cave) failed to identify any offending artery. A vein (1-mm diameter) was lodged between the nerve root and the brainstem. The VO-VREZ compressed the ventral side of nerve root (type A VO-VREZ) and was divided after electrocoagulation. The surgery was completed.

Supplementary Video 2 A representative case of group I, type B VO-VREZ, with relatively large diameter. Exploration of the cistern segment of trigeminal nerve root failed to identify any offending artery. A vein (3-mm diameter) was identified in a position superior and lateral to the exit of the trigeminal nerve root from the brainstem. The VO-VREZ compressed the nerve root (type B VO-VREZ) and was separated from the nerve root, and then interposed with shredded Teflon. The surgery was completed.

Supplementary Video 3 A representative case of typical group II, type B VO-VREZ. Exploration of the cistern segment of trigeminal nerve root identified potential neurovascular conflict between the nerve root and an offending artery, along with a vein (1-mm diameter) in a position superior and lateral to the exit of the trigeminal nerve root from the brainstem (type B VO-VREZ). The VO-VREZ compressed the nerve root. The artery was managed routinely. The VO-VREZ was separated from the nerve root, and then interposed with shredded Teflon. The surgery was completed.

Supplementary Video 4 Second representative case of group II, type A VO-VREZ. Exploration of the cistern segment of trigeminal nerve root identified potential neurovascular conflict between the nerve root with an artery and a vein (1-mm diameter) lodged between the nerve root and the brainstem (type A VO-VREZ). The VO-VREZ compressed the nerve root. The artery was managed routinely. The VO-VREZ was divided after electrocoagulation. The surgery was completed.

Supplementary Video 5 A representative case of group III, type C VO-VREZ. Exploration of the cistern segment of trigeminal nerve root identified a definitive culprit artery at the porus trigeminus of Meckel's cave and a vein (1-mm diameter) that passed between the sensory and motor branches of the trigeminal nerve root (type C VO-VREZ). The VO-VREZ did not compress the nerve root. The artery was managed routinely. The VO-VREZ was left in place. The surgery was completed.
